# An Anniversary Oration on the Subject of Quarantines, Delivered to the Philadelphia Medical Society

**Published:** 1807-08

**Authors:** Charles Caldwell


### *An Anniversary Oration on the Subject of Quarantines, delivered to the Philadelphia Medical Society;* by CHARLES CALDWELL, M. D.

GENTLEMEN OF THE SOCIETY,

THE profession of medicine is, at present, stripped of much of the dark cabalistical covering, which once concealed it from the public eye. The knowledge of it has, long since, ceased to be regarded as a *speciale Dei donum*, a peculiar endowment from above, and is now justly ranked, with other scientific pursuits, among the objects of human attainment. Nor are its most illustrious cultivators any longer venerated as the descendants of the gods. Still, however, it preserves, and is worthy of preserving, a distinguished place in the catalogue of the sciences. It is still far from forming any part of that common stock of information, collected by mankind in their passage thro' life. It is, perhaps, more peculiarly than any other, an insulated profession, and that of such extent and variety, as to be wholly beyond the reach and comprehension of all, all, except its particular votaries; for it is worthy of remark, that no one devoted to other pursuits, has ever become eminent in the science and the profession of medicine. This branch of human knowledge alone, affords matter sufficient for the exercise of the highest talents, applied with unceasing industry, and continued in action during the longest life.

If these observations be true (and I presume their truth will not be controverted) it is peculiarly fortunate that medical subjects, though frequently the topics of general and promiscuous discussion, are but seldom the objects of civil legislation. Circumstances have, however, occasionally occurred, to cause some of them to be acted on by legislative bodies. It is to be lamented that this has, of late years, been the case, in various instances, in our own country. I speak of the event as a matter of regret, because the deliberations on this subject have not only been wholly inadequate to the end in view; but, what is much worse, they originated in error, were conducted without knowledge, and terminated in mischief. If they have not actually increased the very evil they were intended to prevent, they have, at least, without diminishing it, associated it with others of their own creation. They have added another, and I hope a convincing proof, to those before existing, that the science of medical jurisprudence is beyond the sphere of common lawgivers, and that none but medical characters should ever be deputed to its high and interesting concerns.

You no doubt perceive, Gentlemen, that I speak in allusion to the systems of quarantine, established of late by several of our state legislatures, with a view to protect our shores from the ravages of pestilence. Though these establishments were, probably, the result of some reflection, and certainly of the best intentions, on the part of their founders, yet experience has proved them to be erroneous in their principles, and destructive in their tendency. Considered in a national point of view, they are worse—much worse, than the evil they were intended to prevent. Growing, however, as they have done, out of the present state of public opinion relative to the origin and nature of our pestilential diseases, and being perfectly accommodated to that state, nothing short of a general and radical change in this opinion, would be adequate either to abolish or to reform them. But such a change cannot be produced by any single effort, however wise or however powerful. It must be a work of time, and can be accomplished only by a zealous co-operation of many individuals.

But

But to whom shall the important work of effecting this change be entrusted? and what description of characters is best calculated to conduct it to a successful issue? Is it to the lawyer, the statesman, or the divine, that the management and fate of this business should be committed? or, is it not rather to the votaries of medicine, who, from their knowledge of the subject, are alone equal to the difficulty of the task?

To you, Gentlemen, I hope and believe that no inconsiderable part of the honour of this enterprise will belong; and, trust me, it is an enterprise worthy of your ambition, worthy of your exertions; an enterprise not confined in its effects to your own country, nor limited in its duration to the present times; but enfolding in its wide embrace, nations the most distant, and extending to ages the most remote. It is an enterprise which will, in future times, be regarded as the first effectual blow aimed at the existence of that false idol, that seductive Dagon in medicine, which, for nearly four centuries, has commanded the homage of the nations of Christendom.

It is proper, Gentlemen, that you should be duly sensible of your own peculiar fitness, both as to qualifications and opportunities, for co-operating in this important enterprise. Devoted as you are, and must be, by the nature of your profession, to observation and inquiry, you will be naturally looked up to by your fellow-citizens as sources of correct information and wholesome advice, particularly in matters relating to the science of medicine. Established, as you will shortly be, in the practice of your profession, in every part of the United States, you will be capable, from your widely extended influence, of acting, in some measure, on the mind of the whole nation at once. By cultivating, therefore, an unity of sentiment, and by observing a proper concert in your measures and exertions, on any one point, it will be in your power to produce, in relation to that point, somewhat of a national effect. The numerous impressions made by each of you in person, spreading from you individually, as from so many centres, will expand and unite, like adjacent ripples on the peaceful water, till the whole of your fellow-citizens shall feel the impulse. Let the point you may choose to act on be, *the errors into which our state legislatures have fallen, relative to systems of quarantine*, and the evils arising from these systems cannot fail to be done away.

Having then the power to subserve the interest of truth and of your country, on this subject, the exercise of that power must be left to your own wisdom and discretion. I have no doubt, however, but you will be found as prompt and zealous, as you are capable of being efficacious and useful, in your exertions. To convince you of my readiness to co-operate with you, to the full extent of my abilities, and even to expose myself in the front of this war against error and prejudice, I will here submit to your consideration a few remarks on the subject of quarantines. Perhaps a brief view of the origin of these institutions, and of the circumstances attending their first establishment, may aid us in judging of their rationality and usefulness.

The first Lazaretto and system of quarantine, of which we have any account, were established at Venice, in the year 1448, during the ravages of a very destructive pestilence. Not long after this, similar institutions were erected at Marseilles, Genoa, Leghorn, Malta, Messina, Zante, Spezia, and various other European ports, where they have continued, without interruption or any material alteration, to the present day. It must be acknowledged, therefore, that these establishments have the sanction of considerable antiquity to recommend them; and this antiquity has been adduced by some writers as an argument in favour of their great utility, and of the soundness of the principles on which they are founded. Had they not, say these authors, been proved by experience to be effectual and unequivocal guardians of public health, they would long since have fallen into disrepute and been abolished; but this mode of reasoning, though somewhat plausible and imposing, is highly erroneous. Admit antiquity as an infallible test of excellence, and then every institution becomes more valuable, in proportion as it acquires a greater age. In matters where physical science is concerned, the antiquity of establishments, unless they have been frequently altered and amended, is an argument against their excellence rather than in favour of it. The course of science is known to be progressive. As mankind advance in their knowledge of nature, they find it necessary to make frequent changes and innovations in long established opinions and systems, and sometimes to abandon them altogether. This is, perhaps, more particularly the case in things relating to the science of medicine; for I believe it to be true, that medical opinions have undergone more numerous and more rapid changes than those connected with any other branch of philosophy.

If we advert to the circumstances of the *time* in which systems systems of quarantine were first established, we shall derive no argument in favour of them from that quarter. On the other hand, we shall be led to suspect, that they were founded on principles of superstition and prejudice, rather than on those of reason and science. The fifteenth century, which gave birth to these institutions, was a period of physical darkness throughout the world. This was peculiarly the case in Italy, and in the south of Europe in general, where the human mind was led most astray by the delusive wiles of priest-craft, and groaned under the heaviest load of papal tyranny; though at that time, polite literature was cultivated with considerable success in some of the Italian States, particularly at Florence and in other parts of Tuscany, yet Europe does not appear to have been able to boast a single physician or philosopher of real eminence. The whole genius of the age was devoted to warfare, poetry, painting, sculpture, and ecclesiastical learning. As yet, the study of nature, by experiment and observation, the only way in which any progress can be made in physical science, was not only neglected, but wholly unknown. The genius of Bacon, the true father of modern philosophy, had not yet sent forth its illuminating beams. Although a few physicians of that period have transmitted their names and their writings to posterity, yet these writings exhibit little else than a strange discordant mixture of the errors, absurdities, and extravagances of the time. As these writers appear to have had no knowledge whatever of thr real causes of disease, they have indulged themselves in the most unbounded flights of fancy and superstition, in search of imaginary causes; hence they derived some diseases from astral and planetary influence, others from demoniacal influence, and others from the immediate agency of heaven. To this latter class belonged the *pestilence* itself, the very disease for the prevention of which, systems of quarantine were about the same time erected.

At first view, there appears to be, in this particular instance, a striking inconsistency between the doctrines and the practicable establishments of the age; but on a more careful examination of the subject, this seeming inconsistency vanishes. It must be recollected that in the fifteenth century, the fervid enthusiasm, which had previously impelled the hosts of Europe to carry their arms into Asia in the holy wars, had not yet entirely subsided in the breasts of their descendants. The countries of the East continued still to be regarded with mingled emotions of reverence and abhorrence; reverence for the soil which had been rendered sacred by the footsteps and blood of the Messiah, and abhorrence of the idolatrous and impious rites by which that soil was daily polluted. The infidels by whom these abominations were committed, not only throughout the country where the gospel of life had been first promulgated, but even on the hill of Calvary itself, were considered as the proper and peculiar objects of divine indignation. On these heretics therefore, it was believed that the Deity had, by his own immediate act, sent down the destroying *pestilence,* as a punishment for their disbelief of the gospel, and their disregard for the precepts of his Son. Like the late pestilential diseases of the United States, the Asiatic pestilence was *then* erroneously regarded as a *new* disease. But, as it was deemed both new and of supernatural origin, it was considered as also possessed of certain extraordinary properties. Among these was, *its power of being communicated from one country to another.* Although this disease had been, in reality, known from the earliest times, yet, previously to this period, no such power had ever been attributed to it. The fleets and armies of the ancient Greeks and Romans had repeatedly visited Egypt and the other provinces of the East, and returned again to their native countries, without the least restraint or precaution; so had the troops of the various European nations, during the continuance of the holy wars; yet, in these instances, no suspicion appears to have been entertained of the introduction of the Asiatic pestilence into any part of Europe. But we know that this disease raged several times among the crusaders whilst in Asia, as well as in many of the armies of ancient Rome.

Such, then, appears to have been the origin of the doctrine of the importation of pestilence into Christendom. A malignant and fatal disease was believed to have been inflicted as a punishment from heaven, on the infidels of the East. This disease was further believed to be communicated by contagion, through the channels of commerce, to the Christians of the West; and, for the prevention of such a calamity among the latter, systems of quarantine were devised and erected.

But, there was probably another cause which assisted in inducing the inhabitants of Europe, to consider the pestilence as introduced among them from the shores of Asia. This cause also had an indirect relation to the holy wars. The crusaders, on their return from the East, though they were were never charged with the introduction of pestilence, are known to have brought along with them the small-pox, as one of the rewards of their pious expeditions. This circumstance would be likely to induce their descendants, who suffered grievously from that loathsome and destructive complaint, to regard the land polluted by the infidels, as the proper nursery of many other formidable diseases. Having certainly derived one of their calamities from that region, it was not unnatural for them, in their prejudiced and very limited view of things, to look to the same quarter for other evils of a similar nature. Even at the present day, there are certain strange and irrational notions entertained on this subject. The countries of Asia are still considered by many as the birth-place of particular diseases, which cannot originate in Europe or America, although the climates of these several regions are, in many places, precisely similar, and all their other physical causes are capable of operating with the same degree of force. Such sentiments, like the dreams of the physicians of the fifteenth century, are at open war with the very rudiments of philosophy.

There is still a further circumstance, which also contributes to give a very superstitious complexion to the origin of quarantines. The term itself imports, that *forty days* constituted the period of time, deemed necessary to be set apart for the purification of things infected. But the adoption of this period cannot be considered as the result of a philosophical inquiry, relative to the nature of pestilential poison, and the space of time requisite for its removal or destruction. It is derived merely from a superstitious regard for the number *forty*, on account of the accidental relation which that number bears to certain events and circumstances recorded in the Old and New Testaments. Thus, the Israelites were *forty* years in traversing the wilderness between Egypt and the Land of Promise. Under the Laws of Moses, the term of *forty* days was necessary for the completion of certain purificatory processes. Christ fasted *forty* days in the wilderness, for the purpose, as some commentators allege, of purifying his body from all such passions and propensities, as might tend to render it disobedient to the dictates of the spirit. Therefore, said the priests, and other superstitious zealots of the day, *a quarantine*, or lustration of forty days, is requisite for the cleansing of vessels, and other articles, contaminated by pestilential contagion.

Such, then, were the time when, and the circumstances under which, lazarettoes and systems of quarantine were first established by the nations of Europe. The age was a period of great darkness, and the whole business appears to have originated in, and to have been deeply tinctured by, the religious bigotry and delusion of the times. As a further evidence of this bigotry, it may be observed, that lazarettoes and pest-houses were, at first, entrusted almost exclusively to the care of ecclesiastics, whose piety and sacred character were supposed to be a shield against the arrows of contagion. Nor have the rules, regulations, and general management of these institutions undergone any very material amendment or alteration, even down to the present day. In the south of Europe, lazarettoes and quarantine establishments are in nearly the same condition, and under nearly the same government now, that they were three hundred years ago. The light which, like a day star, has burst forth in all departments of physical science, does not appear to have penetrated, as yet, the ancient night of these establishments. They are, in almost as high a degree, overwhelmed by prejudice, superstition, and error, at present, as they were at the time of their first institution. Yet have they served as the chief models for similar establishments in most other parts of Europe. Hence, when the celebrated Howard set out on his travels to acquire a knowledge of quarantines for the benefit of his country, he directed his course to the shores of the Mediterranean. Yet, from the account which that writer has himself given of the several pest-houses and lazarettoes, which he visited in Italy and elsewhere, they appear to the eye of philosophy, much better calculated to generate than to prevent pestilential diseases; for they are mere dungeons of dampness, filth, and putrefaction. The ceremonies through which all persons and articles arriving at them from sickly or suspected places are obliged to pass, are as unmeaning and preposterous as any of the rites of the most superstitious form of worship. Indeed, such is the perverted state of these establishments, that (supposing their continuance to be necessary at all) it calls for the reforming hand of a medical Luther, or a Calvin, no less than the abuses of the Church did during the pontificate of Leo X. If circumstances do not even demand an entire demolition of them (which I am persuaded they do) they demand, at least, their complete regeneration.

In reply to these observations, it will be probably said, that that the systems of quarantine, against which I am here inveighing, have (notwithstanding my charges of error and superstition) had the happy effect of preserving Europe from the Asiatic pestilence. Were this indeed the case, my arguments against them could have but little weight. The assertion, however, is wholly unfounded; they never protected a single individual, much less a whole country, from this horrible disease. For nearly two centuries after the establishment of these systems, the south of Europe was visited by pestilence, as frequently as it had ever been before. Its exemption from that calamity, in latter times, has been owing to causes that have no connexion with lazarettoes and quarantines. These causes consist in the agricultural improvements which have drained and dried up offensive marshes and large bodies of stagnant water, in the introduction of forms of police, enforcing greater cleanliness and purity in large commercial cities, and in a radical change in the customs and modes of living of the inhabitants. In other words, they consist in the removal of extensive sources of septic exhalations, in higher degrees of personal and domestic cleanliness, and in the adoption of a diet and mode of life, better suited to the nature of the climate, and the constitution of the people. Let Italy and other countries of the south of Europe be reduced to the half-cultivated state in which they were during the fifteenth, sixteenth, and seventeenth centuries, and they will again, notwithstanding their quarantines and lazarettoes, be subject as before, to pestilential epidemics. On the other hand, let even the countries of Asia be put under a proper state of agricultural improvement, let their cities become subject to a wise and well executed police, and let their inhabitants live in a manner accommodated to the climate, paying in particular, a due regard to personal and domestic cleanliness, and pestilence will cease to be the perpetual opprobrium and scourge of the East; for there is no country on earth so abandoned by nature to inevitable calamity, as to be *necessarily* subject to that disease.

Very early in the eighteenth century, the pestilence had been slightly epidemic in several of the countries of Europe. At length, in the year 1720, it hroke out in Marseilles, where it prevailed for a time with unbridled rage and great mortality. Believing the disease to be highly contagious, Great Britain began to tremble for her safety. Accordingly, the eye of the British ministry became eager in search of means to avert from the nation the im-

 pending pending evil. In this state of apprehension and anxiety, application was made to Dr. Meade, physician to Geo. II. who, if not the most able, was at least the most popular physician of the kingdom. This favourite of the court was requested to furnish a system of rules and regulations to prevent the introduction of a disease which was committing such ravages in the south of France.

In complying, or rather in attempting to comply with this request, Dr. Meade had nothing but his knowledge of books to direct him. He had had no personal experience in pestilential diseases, and could, therefore, do nothing else than simply tread in the footsteps of his predecessors. We accordingly find him immersed in most of the weaknesses and errors, and even in some of the wild extravagancies of the dark ages. He did not indeed contend, that pestilence was an engine of vengeance launched immediately from the hand of the Deity; he considered it as an evil of terrestrial origin. But he was firmly of opinion that it had been always generated in the sultry climates of the East, and introduced from thence into Europe by means of contagion. Respecting the virulence and activity of this contagion, he relates several stories too gross and extravagant even for the ear of credulity itself. It would be unpardonable, therefore, to offend your ears by a repetition of them.

As to Dr. Meade's practical rules and directions on the subject of quarantine, he candidly acknowledges that they are derived entirely from the practices long pursued in the south of Europe. He does not even attempt the suggestion of a single improvement as to the mode of preventing the introduction of pestilence from foreign places. He has indeed left both the business of quarantine and the doctrine of contagion precisely as he found them, except that he impressed the errors which he had himself imbibed respecting them more deeply than before on the minds of his countrymen. For, possessed, as he was, of eloquence, ingenuity, and address, and sanctioned in his efforts by national and royal patronage, he spoke and wrote with the weight of an oracle. Such was for a time his ascendancy over the public mind, that it was deemed a kind of medical heresy to dissent from his opinions. I fear, that even at the present day, the sentiments of Dr. Meade, respecting pestilential contagion, have a secret yet powerful influence over the minds of many physicians, both in England and the United States. Had he never written on contagion and quarantines, error on these subjects would never have been been able to boast such a widely extended and such a protracted reign.

From the time of Dr. Meade, till within the last thirteen years, but little attention was bestowed upon the business of quarantines. The cities of Europe remained free from pestilence, and no event took place to bring the subject into notice. In consequence of this, the principles and practices long established in these institutions, continued in use without question and without alteration. Lazarettoes and systems of quarantine were the only institutions bearing any relation to science, which had been handed down from the fifteenth to the eighteenth century, without having undergone either revision or amendment. They were still, therefore, deeply tinctured with the superstition and semi-barbarism of the age in which they were founded.

It came at length to the turn of the United States to figure in the great drama of contagion and quarantine. Her part was a tragical one; and it must be confessed, that she has performed it with such effect as to overwhelm both science and humanity in tears.

The pestilence appeared first in the city of New-York, in the year 1792. But as it was confined within narrow limits, and produced but little mortality, it did not become an object of much public notice or solicitude. In the year 1793, the same disease, but very different in violence and malignity, broke out and raged in the city of Philadelphia. The sudden and great mortality which it occasioned, and the unprecedented rapidity with which it spread among the inhabitants, struck both the city and the country with the utmost consternation. Even the most distant parts of the United States, seemed at first to tremble for their safety. The disease was admitted by every one to be highly contagious, and was very generally believed to have been introduced into Philadelphia by a sickly vessel from the island of St. Domingo. This belief, which, without the slightest examination into circumstances, was hastily adopted, and propagated with a kind of apostolic zeal, has proved a source of the most grievous misfortune to the commerce and prosperity of our city.

To the honour of our country, however, this opinion was not universal. When the pestilence appeared in Philadelphia in the year 1793, Dr. Rush, supported by a few other physicians, boldly declared it to have originated from local and domestic causes. But, like the still, small whispers of conscience amid the boisterous uproar of the passions, the voice of these interpreters of Nature was drowned drowned at the time by the loud and general cry of "*importation from abroad!*" Accordingly, systems of quarantine, founded on a presumption of such importation, were soon afterwards established at Philadelphia, New-York, Baltimore, and other commercial cities of the Union. They were directed particularly against vessels arriving from the West-India islands, which were regarded, if not as the birth-place, at least as the nursery of the evil which such systems were intended to prevent. These establishments were nothing more than mere copies of similar institutions in the old world. Indeed, as they were erected in great haste, and under the direction of men who had never before reflected on the subject, they could not be expected to assume an improved form. Nothing in their organization bespoke either the superintendance of sound reason, or an acquaintance with nature. They appeared in all their departments to be the offspring of mere chance, or of something more systematically erroneous. Directed exclusively against *contagion*, supposed to be in some way attached to vessels arriving from tropical climates, they paid at first no regard to the real *cleanliness* of the vessels themselves. Filth of every description appeared to be regarded by their framers as an innocent article. This was more particularly the case with respect to the system of quarantine established in the port of Philadelphia.

The insufficiency of these establishments for answering the end proposed, was soon discovered in every quarter. Notwithstanding the strictness with which the measures they enjoined were executed, the pestilence appeared repeatedly in most of our large commercial cities, as well as in various inland places. This produced frequent changes and modifications in our systems of quarantine, which it would be alike impracticable and useless to specify. They had all the appearance of mere experiments, made by undiscerning men, without any established principles to direct them. Unfortunately for the importers of pestilence, the result of each succeeding experiment proved alike unfavourable to their opinions and expectations. Notwithstanding this, the establishment was still kept up, and the confidence of many in the efficacy of quarantines remained unshaken. The perpetual cry of these characters was, "Let our quarantines be so strict and rigid in principle, and so faithfully executed, as to suspend all intercourse with the West Indies and other tropical climates, during the summer and autumnal months, and the calamity of pestilence will cease to afflict us."

But

But I perceive, Gentlemen, that I have already trespassed too much on your time. I will, therefore, pursue the history of quarantines no further. I flatter myself I have dwelt on the subject sufficiently at large, to give you a view of its most prominent features. I have already stated to you, and I beg leave to repeat it, that the practice of quarantines commenced during the dark ages, when Europe was a stranger to physical science; that it appears to have owed its origin, in a great measure, to a superstitious abhorrence entertained towards the infidels of the East; and that it has been continued down to the present day, without any material alteration or improvement. While other practices and institutions of similar date have been either abolished or amended, in consequence of the light shed on their principles, by the advancement of science in modern times, this alone has been suffered to remain immersed in its primitive darkness, and surrounded by all its original errors.

What have been the effects of quarantines on the interest and prosperity of the United States? Have they, in a single instance, been instrumental in protecting any one of our sea-ports from the ravages of pestilence? We owe it to truth and independence of sentiment, we owe it to our country, to assert that they have not. Those commercial cities, in which no quarantines have existed, or in which they have been so light as to exist only in name, have been as free from pestilence as others where they have been practised in their utmost rigour. Of the truth of this, Philadelphia furnishes a melancholy example. For twelve years past has her commerce languished under the weight of the fetters of quarantine, while that of other principal cities of the Union has been comparatively free. Yet has she not, from this state of severe and oppressive restraint, derived the shadow of advantage on the score of health. With the single exception of New-York, which has experienced equal calamities, she has been by far the greatest sufferer in the Union from pestilential visitations. Her citizens have suffered incalculably in their fortunes, without being rewarded by any equivalent in the security of their persons. A few years ago, Philadelphia stood proudly pre-eminent on the scale of commerce. But what is now her humiliated state? Stripped of her ancient and well earned pre-eminence, she beholds herself sunk to a secondary rank, and that by the sole operation of an unwise and destructive quarantine.

But,

But, though Philadelphia has been most deeply affected in her interests, yet the commerce of the United States at large has suffered severely from systems of quarantine. We have declared, as a people, by our public acts and public writings, that our pestilential epidemics are highly contagious, and capable of being conveyed from country to country. The nations of Europe have taken us at our word, and assumed, with respect to us, a defensive attitude. During certain months in the year, our vessels are prevented from entering their ports, except after the performance of an oppressive quarantine.

For this evil we are indebted entirely to our own ignorance and indiscretion. We are ourselves the cause of our being regarded abroad as an infected people, with whom, at certain seasons, it is dangerous to hold an immediate intercourse. In the West-Indies as well as in the tropical parts of South America, the inhabitants are subject to pestilential diseases precisely such as we experience. Yet neither in England, France, Spain, Portugal, nor Italy, are vessels from those burning and sickly regions subject to quarantine. Ever since the discoveries of Columbus have such vessels been admitted into the ports of those countries, without restraint, and without even the suspicion of introducing contagion.

Whence, then, arise those rigorous measures of quarantine, which are pointed exclusively against vessels from the United States? Why are vessels from the West-Indies and the tropical section of Spanish America suffered to enter the ports of Europe without molestation, while ours are prohibited under the heaviest penalty? The answer to this is obvious and easy.

The inhabitants of the West-Indies and of South America declare unanimously, that the pestilential diseases of those regions are not contagious, and, therefore, not capable of being transported to the mother country. We, on the other hand, declare the reverse to be true, with regard to the pestilence which has prevailed in our commercial cities. The governments of Europe, giving full credit to both our assertions, and framing their measures accordingly, very consistently subject to quarantine, at certain times, all vessels arriving from the United States, while those from the West-Indies and South America are suffered to pass without detention. Had we not, with an equal want of truth and of wisdom, attributed to the pestilence of our country a quality which it certainly does not possess (I mean contagion) whatever our sufferings might might have been from it at home, they would not have been doubled by extending to our commerce in foreign places. On the other hand, should the inhabitants of the West-Indies, even at this time, pronounce their febrile diseases to be contagious, there can be no doubt but the nations of Europe would take the alarm, and include in their measures of quarantine all vessels from those regions. Such are the consequences which every people must expect, who proclaim their country to be a nursery of contagion.

When compared with each other, the practices of quarantine in Europe and in the United States, exhibit a strange and ludicrous inconsistency; an inconsistency disgraceful to nations which call themselves enlightened. Thus, in the United States, we subject to quarantine, vessels arriving from the West-Indies, pronouncing our own country free from pestilence, unless when introduced from that quarter. In Europe, on the other hand, all vessels from the West-Indies are exempt from quarantine, while those from the United States are compelled to submit to it. In other words, we declare the West-Indies to be the genuine birth-place of pestilential contagion, which gains admission into our own country only through the channels of commerce. But the nations of Europe deny this, attaching the opprobrium of contagion exclusively to the United States, and contending that the West-Indies are free from it. Such inconsistencies in practice bespeak a radical error in principle.

But we have, hitherto, dwelt only on the dark and cheerless side of our subject. Let us, for a moment, direct our views to that quarter of it, where a more bright and pleasing prospect invites us; that quarter, where the dawn of a new era, in medical science, has already unbarred its ruddy portals. On the front of these portals, behold inscribed, in shining letters, the sublime and prophetic effusion of the poet.

"*Magnus ab integro sæclorum nascitur ordo.*"

A new series of events is about to commence their brilliant course.

Yes, Gentlemen, as far as relates to pestilential diseases, a new series of events is, indeed, about to commence; I should rather say, it has already commenced, and is destined to abolish the doctrine of pestilential contagion, and with it all pest-houses, and systems of quarantine throughout the world. Let it be our pride to remember, that this this series first began its course among ourselves, and is now forcing its way through the nations of Europe. It is several years since all the most enlightened physicians of the United States rejected the doctrine of contagion, as far as relates to the pestilence of our own country. These characters contended of course, that this disease was not, and could not be, imported from abroad, and that, therefore, systems of quarantine for its prevention were useless establishments. This opinion has found its way into the South of Europe, and has been ably supported, as applicable to the pestilential diseases which have lately prevailed in that quarter. The most enlightened physicians of Spain and Italy have contended and proved, that these diseases were neither contagious nor imported from abroad, and that, therefore, systems of quarantine were useless and nugatory, as means of prevention.

But it may be asked, shall there, then, be no quarantine establishments in the United States? and shall all vessels from tropical climates, whether healthy or not, be suffered at all seasons of the year to enter our ports immediately on their arrival? I answer, let the detention of quarantine be entirely abolished, and the process of simple *purification* substituted in its stead. This process consists solely in washing and ventilating vessels, and may be completed in three days as well as in forty. All vessels, after long voyages, whether sickly or not, and from whatever place or climate they may have come, ought, during warm weather, to be compelled to undergo this necessary purification, previously to their admission into any of our ports. Such a measure is not too rigid for commerce to bear, and it is all that the public safety requires.

But it is not alone with regard to the pestilential diseases of Europe and America, that the doctrine of contagion is falling into disrepute. The same enlightened sentiment begins to prevail relative to the plague of Asia and Egypt. It is six years since I had the honour, in this hall, of addressing your predecessors in our society, on the analogies between the pestilence of the East and the yellow fever or pestilence of the West. I contended at that time, though in opposition to an unbroken host of prejudices, that *neither* of these forms of disease is contagious. And I now with sincerity assure you, that as my years accumulate and my observation extends, my conviction of the truth of this opinion increases in strength. But, what is still more consolatory and encouraging, I have the happiness to find, that the same opinion (which is becoming popular in some some parts of the United States) is also advocated by many distinguished characters in other countries.

In contemplating this particular head of our subject, we find reason to rejoice, that even war itself is not at all times an unqualified evil. Amidst its varied and destructive operations, some good occasionally breaks forth, to make partial amends for its horrors and calamities. The armies, on their return from the holy wars, during the dark ages, are known to have brought with them from the countries of the East, the semina of those arts and sciences, which have since taken root, and enlightened and aggrandized the nations of Europe. In like manner, the late French and English expedition into Egypt has been the cause of diffusing through Europe and America much valuable information relative to the oriental pestilence. The ablest of the physicians and philosophers who attended the respective armies on that occasion, have ascertained, and proved in their writings, that this disease is not contagious. They have clearly shown it to be nothing but the endemic of the eastern climates, depending for its origin and existence on local causes, and wholly incapable of being conveyed and propagated in distant countries. As soon as this sentiment shall become general among the nations of Europe, (and, being true, it must become general) their systems of quarantine for the prevention of the Asiatic pestilence will be regarded as useless, a sure precursor of their final demolition. Indeed, if we carefully examine the writings of Bruce, Volney, Sonnini, Antes, and other travellers, they cannot fail to convince us, in opposition to the avowed sentiments of their authors, that the plague of Egypt and Asia is not contagious. Even the treatise of Russel himself, the great apostle of contagion, contains facts sufficient to refute his own doctrine.

If, then, Gentlemen, such is the nature and such the tendency of systems of quarantine; if these systems are indeed founded in error and superstition, and productive only of mischief, you are called on by your love of truth, and by that spirit of patriotism, which it is, no doubt, your delight, as it is certainly your duty to cherish, to aid in the demolition of those of the United States. It is known to you that the quarantine establishments of our country are bottomed on special laws enacted for the purpose. While these laws continue in force, it would be criminal to impede their operation or frustrate their intention. They spring, however, like all others, out out of the general sentiment and will of the people. To strike effectually at the root of the evil, this sentiment, which is now erroneous, must be corrected, and this will, which is now misguided, must be made to assume a proper direction. In other words, the people must be made to think correctly on the subject of quarantines, and a repeal of the laws on which these institutions are founded will be the immediate consequence. To you, Gentlemen, I beg leave to repeat, this work of reform in the public sentiment must be, in part, entrusted. Your influence in society, on this subject, will be extensive and weighty. On precisely the same scale then will be your duty to act. Let it be your constant endeavour to use this influence in such a way, as to convince your fellow-citizens, that the pestilence of our country is neither imported from abroad nor contagious in its nature, and that, therefore, our quarantine establishments, intended for its prevention, are useless and oppressive. This service, not only your country in particular, but your contemporaries at large, have a right to expect from you, and posterity will regard your memory according as you perform it.

I should be wanting in respect for the interesting occasion on which we are convened, were I to part from you without tendering you my sincere congratulations on the uncommonly flourishing state of our Society. The clouds of schism, which hung over it of late, and the breath of adversity, which threatened to blast it, have only served to renovate its vigour. On these temporary evils it may now look back with emotions of triumph. Intimately connected as it is with the University of Pennsylvania, possessing funds that will shortly be sufficient for all its purposes, and firmly rooted, as to its welfare, in the affections of its members throughout the Union, it may be confidently said to have but little to wish, and nothing to fear. Nor can it fail, with such advantages, and under proper management, to attain and preserve an elevated rank among the medical associations of the age. Let these considerations inspire its friends with higher zeal, and invigorate their efforts for its support and promotion. And let me hope, Gentlemen, that each of you will be prepared to reciprocate the sentiment, with sincerity and becoming fervour, when I say of our society, " *Esto perpetua!*

